# Carotid blowout syndrome in patients treated by larynx cancer^☆^

**DOI:** 10.1016/j.bjorl.2016.08.013

**Published:** 2016-09-29

**Authors:** Carlos Miguel Chiesa Estomba, Frank Alberto Betances Reinoso, Alejandra Osorio Velasquez, Olalla Castro Macia, Maria Jesus Gonzalez Cortés, Jesus Araujo Nores

**Affiliations:** University Hospital of Vigo, Otorhinolaryngology, Head and Neck Surgery Department, Pontevedra, Spain

**Keywords:** Carotid, Blowout, Larynx, Cancer, Carótida, Ruptura, Laringe, Câncer

## Abstract

**Introduction:**

Carotid blowout syndrome is an uncommon complication for patient treated by head and neck tumors, and related to a high mortality rate.

**Objective:**

The aim of this study was to study the risk of carotid blowout in a large cohort of patients treated only by larynx cancer.

**Methods:**

Retrospective analysis of patients older than 18 years, treated by larynx cancer who developed a carotid blowout syndrome in a tertiary academic centre.

**Results:**

197 patients met the inclusion criteria, 192 (98.4%) were male and 5 (1.6%) were female. 6 (3%) patients developed a carotid blowout syndrome, 4 patients had a carotid blowout syndrome located in the internal carotid artery and 2 in the common carotid artery. According to the type of rupture, 3 patients suffer a type I, 2 patients a type III and 1 patient a type II. Five of those patients had previously undergone radiotherapy and all patients underwent total laryngectomy. We found a statistical correlation between open surgical procedures (*p* = 0.004) and radiotherapy (*p* = 0.023) and the development of a carotid blowout syndrome.

**Conclusion:**

Carotid blowout syndrome is an uncommon complication in patients treated by larynx tumours. According to our results, patient underwent radiotherapy and patients treated with open surgical procedures with pharyngeal opening have a major risk to develop this kind of complication.

## Resumo

**Introdução:**

A síndrome da ruptura da carótida é uma complicação incomum no paciente em tratamento para tumores de cabeça e pescoço, relacionada com uma alta taxa de mortalidade.

**Objetivo:**

O objetivo deste estudo foi estudar o risco de ruptura da carótida em uma grande coorte de pacientes sendo tratados isoladamente por um câncer de laringe.

**Método:**

Análise retrospectiva de pacientes com mais de 18 anos, tratados por câncer de laringe em um centro de assistência terciária, que desenvolveram a síndrome da ruptura da carótida.

**Resultados:**

Ao todo, 197 pacientes atenderam aos critérios de inclusão, 192 (98,4%) eram do sexo masculino e 5 (1,6%) eram do sexo feminino. 6 (3%) pacientes desenvolveram síndrome da ruptura da carótida, 4 tiveram síndrome da ruptura da carótida localizada na artéria carótida interna e 2 na artéria carótida comum. De acordo com o tipo de ruptura, 3 pacientes apresentaram síndrome da ruptura da carótida tipo I, 2 pacientes, síndrome da ruptura da carótida Tipo III e um tipo II. Cinco desses pacientes haviam sido previamente tratados com radioterapia e todos os pacientes foram submetidos a laringectomia total. Encontrou-se uma correlação estatística entre procedimentos cirúrgicos abertos (p = 0,004) e radioterapia (p = 0,023) e o desenvolvimento de síndrome da ruptura da carótida.

**Conclusão:**

A síndrome de ruptura da carótida é uma complicação rara em pacientes tratados para tumores de laringe. De acordo com nossos resultados, pacientes submetidos a radioterapia e pacientes tratados com procedimentos cirúrgicos abertos com abertura da faringe apresentam um risco maior de desenvolver essa complicação.

## Introduction

Carotid blowout syndrome (CBS) is an uncommon complication for patients treated by head and neck tumours (HNT).^1^ The incidence of carotid blowout in patients who underwent surgical procedures involving head and neck cancers ranged from 2.9% to 4.3%.^2^, ^3^, ^4^, ^5^ In those who received re-irradiation because of recurrent head and neck cancers, the incidences of carotid blowout varied from 2.6% to 10%.^6^, ^7^ In this way, CBS is more frequent in patients with HNT and those cases when radiation induced necrosis, recurrent tumours, wound complications from neck dissection, or vessel erosion from pharyngocutaneous fistulas.^8^

The mortality rate of carotid blowout was reported to range from 3% to over 50% in the literature.^3^, ^4^, ^5^, ^7^, ^8^, ^9^, ^10^ Therefore, in a recent meta-analysis, the mortality rate of carotid blowout after re-irradiation in those patients treated by head and neck tumours was as high as 76%.^6^ On the other hand, the neurological sequela reported in those patients who survived an acute episode of carotid blowout, was from 16% to 50%.^9^

In the past, the traditional approach to treat this kind of complication was the surgical revision or ligation.^4^, ^5^ However, these tendencies have changed in recent years into a less aggressive approach, and nowadays, endovascular techniques, including balloons, destructive (embolization) and constructive (stent grafting) techniques, performed by interventional neuroradiologists are gaining popularity and having promising results.^1^, ^9^, ^10^

Few studies have discussed the relevant risk factors of carotid blowout occurred in patients treated by head and neck cancer in general. However, the aim of this research was to study the risk of carotid blowout in a large cohort of patients treated only by larynx cancer.

## Methods

A retrospective analysis was performed on previously untreated patients, diagnosed with squamous cell carcinoma (SCC) of the larynx (cT1-cT4), N−/+, M−/+ according to criteria of the Union Internationale Contre le Cancer (UICC) and the American Joint Committee on Cancer (AJCC) between January of 2009 and January of 2012. Identification of cases was achieved by informatic research on the medical records of our database, using the International Classification of Diseases (ICD-9). This study was approved by the ethics committee of our centre. The demographic data (age, sex), past medical history, comorbidities, stage, type of surgery, CBS as a complication, were obtained by a review of medical history.

Prior to surgery, all cases were discussed in an interdisciplinary committee of head and neck tumours. Preoperative examination of the lesion was carried out by indirect laryngoscopy, videolaryngoscopy and CT scan or MRI of the neck to evaluate the cartilage, preepiglottic and paraglottic space and lymph node involvement. Fine needle biopsies of the neck nodes were performed usually under ultrasonographic control. Those patients with lesions suspicious of malignancy were scheduled for laryngeal microsurgery and panendoscopy with biopsies, followed by a total laryngectomy (TL), horizontal partial laryngectomy (HPL), transoral laser microsurgery (TLM), and radiotherapy alone or accompanied with chemotherapy (QT), in those cases where malignancy was present.

Additional postoperative radiotherapy was administered to some patients with advanced neck disease (N2a/b/c, N3), when the histopathological examination revealed extracapsular spread or, in those patients with lymphatic micrometastases. Patients with histologically close surgical margins, mainly at the base of the tongue, also received post-surgical radiotherapy 4 weeks postoperatively followed by weekly doses to reach a total dose of 60 Gy.

The type of rupture was classified according to Powitzky et al.^11^ Type I: “Threatened” include all those CBS who occurs when the carotid artery is exposed through soft tissue breakdown, secondary to mucocutaneous fistula, infection, tissue necrosis, recurrent tumour or a combination of these. Type II: “Impeding”, when the rupture was limited, it could be temporarily solved with pressure and wound packing and preceding the ultimate haemorrhage by a period of months, and type III “Active or Rupture” is considered rapidly fatal.

All patients with suspect of CBS were evaluated and several patients with type I and II CBS lesions were treated by neuro-radiologist, and type III was treated with surgery. Carotid blowout was confirmed by possible causative lesions, including endoluminal irregularity or disruption, pseudoaneurysm formation, and extravasation.^11^ Patients who had an acute bleeding but who did not receive angiographic examinations were not considered to have had a carotid blowout. Risk for cerebral ischaemia was determined by balloon occlusion test underwent embolization. Those patients who could not tolerate this were considered for carotid stenting.

In our department, patients treated for head and neck malignant tumours are followed during 5 years at least. However, for this study, we considered a group of patients that have been followed for a minimum of 36 months.

Statistical analysis was run in SPSS program for Windows, Version 20.0 (SPSS, INc. Illinois, EE.UU). Quantitative variables in the study were expressed as media ± typical deviation. The different variables were correlated by Pearson Chi-square test and for the comparison of continuous variables. Values of *p* < 0.05 were considered to be statistically significant in all tests.

## Results

197 patients met the inclusion criteria, 192 (98.4%) were male and 5 (1.6%) were female. The mean age was 63.8 ± 10.13 (Min: 40/Max: 88). Of these 37 (18.5%) were diabetics and 69 (34.5%) were hypertensive. The patients had a mean postoperative haemoglobin level of 12.7 ± 1.89 g/dL and a mean albumin level of 41.0 ± 2.98 g/L. Tumoural stage of patients included 39 (19.7%) as stage I, 39 (19.7%) as stage II, 53 (26.9%) as stage 3 and 66 (33.5%) as stage IV. 138 (70.05%) patients were classified as N0, 14 (7.1%) as N1, 13 (6.5%) as N2A, 16 (8.1%) as N2b, 13 (6.5%) as N2c and 3 (1.5%) as N3. There were 4 (2.03%) cases of distant metastases (M1). The mean follow-up was 46.1 ± 12 months (Min: 11/Max: 72). Regarding the type of surgery, the most common was the transoral laser microsurgery for glottis tumours (58 = 29.44%) and total laryngectomy without chemo-radiation therapy (23 = 11.6%) (Table 1).

**Table 1:** Demographic data of patients with larynx cancer with and without carotid blowout.

Variable	Totals (%)	CBS cases
*Age*	63.8 ± 10.13 (Min: 40/Max: 88)	
*Sex*	M: 192/F: 5	
*Mean follow-up was*	46.1 ± 12 months (Min: 11/Max: 72)	
*Post-Op HgB level*	12.7 ± 1.89 g/dL	
*Albumin level*	41.0 ± 2.98 g/L	

*T stage*		
I	39 (19.7%)	
II	39 (19.7%)	1
III	53 (26.9%)	1
IV	66 (33.5%)	4

*N stage*		
N0	138 (70.05%)	
N1	14 (7.1%)	
N2a	13 (6.5%)	
N2b	16 (8.1%)	
N2c	13 (6.5%)	
N3	3 (1.5%)	

*M stage*		
M0	193 (98%)	
M1	4 (2%)	

CBS, carotid blowout syndrome.

Six (3%) patients treated by laryngeal cancer developed a CBS (Table 2), 4 patients had a CBS located in the internal carotid artery (ICA) and 2 had a CBS located in the common carotid artery (CCA). According to the type of rupture, 3 patients suffer a type I, 2 patients suffer a type III and 1 patient suffers a type II. 5 of those patients had previously undergone radiotherapy and all patients underwent total laryngectomy. However, anyone underwent a radical neck dissection (Table 2).

**Table 2:** Demographic data of patients with larynx cancer and carotid blowout.

Variable	Total (%)
Age	62.5 ± 13.48 (Min: 49/Max: 79)
Sex	M: 5/F: 1
Mean follow up	17.8 ± 20 months (Min: 1/Max: 56)
Post-Op HgB level	10.2 ± 1.68 g/dL
Albumin level	2.98 ± 1.56 g/dL

**Figure 1:**
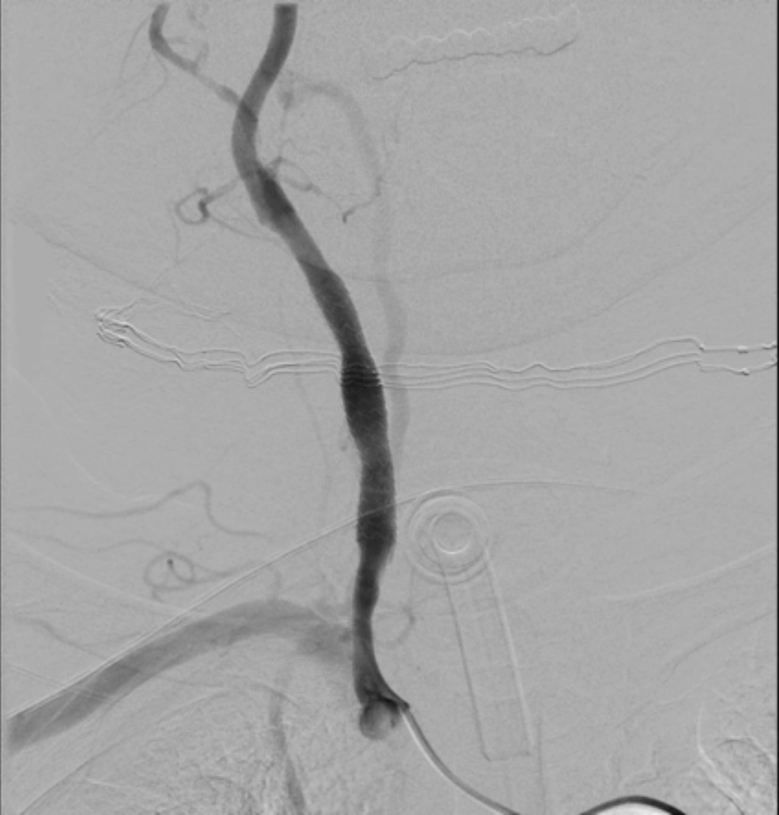
Left carotid artery stent in a patient who suffer a carotid blowout syndrome.

About the cause of CBS, 3 patients suffered radiation induced necrosis proved by pathological and image study, 2 patients present vessel erosion from pharyngocutaneous fistulas and tumour recurrence was proved by pathological examination in 1 patient. Nonetheless, 2 cases were managed with embolization, 1 case was managed with surgery and one patient was treated with a stent (Fig. 1). The other 2 patients died due to severe bleeding in the emergency room. Neurological sequelae were evident in 2 patients due to cerebral stroke, 1 patient after ligation and the other one after ICA embolization (Table 3).

**Table 3:** Patient treated by carotid blowout syndrome at our institution.

Sex	Treatment	Stage	Cause of rupture	Side	Site	Type	Neurological complaints	CBS treatment
Male	TL + CND + QT + RT	T4aN0M0	Necrosis	Right	ICA	I	No	Embolization
Male	TL + CND + QT + RT	T2N2cM0	Fistula	Left	ICA	III	Die	Die
Male	TL + CND + RT	T4aN2bM0	Necrosis	Left	CCA	I	No	Stent
Female	TL + CND + RT	T3N1M0	Necrosis	Right	ICA	III	Die	Surgery
Male	TL + CND	T4aN2cM0	Fistula	Right	CCA	II	Yes	Embolization
Male	TL + CND + RT	T4aN0M0	Tumour erosion	Left	ICA	I	Yes	Die

## Discussion

CBS can be considered as an iatrogenic complication of HNT treatment. The syndrome was described at first in 1962, since then, several surgical and endovascular treatment options have been attempted.^12^ At the beginning, CBS could be treated only with surgical ligation or surgical bypass of the carotid artery. However, these techniques were associated to high mortality and to high neurologic morbidity with rates about 40% and 60%, respectively.^13^ In the mid of 80s, endovascular techniques to managed acute CBS were introduced.^14^ Then, this treatment has gradually gained popularity due the ease of the approach and lower morbidity and mortality rates compared to the surgical approach.^1^, ^13^, ^15^

Short and long term effects of radiation over arteries have been reported. A total radiation doses of 40 Gy over a 10 day duration could induce damage to the vasa vasorum of large arteries and it might be related to the rupture of great arteries in dogs according to McCready et al.^16^ Free radicals produced by radiation were also found to cause thrombosis and obliteration of vasa-vasorum, adventitial fibrosis, premature atherosclerosis, and the weakening of the arterial wall in the histological examination of resected carotid arteries.^10^, ^11^, ^17^ We also found a significant statistical difference in the appearance of CBS, in those patients who received RT treatment before surgery (5/6 = 83.3%) (*p* = 0.023) (Table 4).

**Table 4:** Statistical analysis of factors commonly associated with carotid blowout.

Variable	*p* = 0.05
RT	0.023
Neck dissection	0.151
Open surgery	0.004
Fistula	0.842

Furthermore, some authors suggest the underestimated role of infections in CBS (tissue necrosis or fistula), and the relation of bacterial inflammation as a cause of vasa vasorum thrombosis, and secondary arterial wall damage.^18^ This is why summarizing the effects over vasa-vasorum of radiation and infection, it is necessary to take into consideration the importance of these factors, due to the adventitial layer, which carries about 80% of the blood supply to the remaining walls of the carotid artery. In our series of patients affected by CBS, 2 (2/6 = 33%) patients suffered a pharyngo-cutaneous fistula in the early post-operative period, and 3 (3/6 = 50%) other patients suffered radiation inducing tissue necrosis.

Neck surgery is another significant factor related to CBS, because this type of surgeries could compromise the nutrition of the carotid artery during cervical nodes resection, resulting in injury to the adventitial layer, and this deleterious effect occurs independently of radiation.^9^ Radical neck dissections render the carotid artery more vulnerable to rupture because of the lack of supporting healthy tissues.^11^ Moreover, in those patients with accompanying pharyngeal surgery, there is a higher risk to develop a CBS due to major proportion of salivary fistula formation^6^ and when a hemithyroidectomy has also been carried out, the carotid artery lies very close to the skin and the tracheal stoma increasing the risk of damage over the artery. In relation to larynx tumours, Chen et al. found an incidence of 0.9% of CBS in patients treated by larynx tumours, a lower percentage compared with our results.^19^ However, previous literature review reports the larynx as the most common primary tumour site in almost 23% of patients who suffered a CBS.^11^

In our series, we only included all those patients who came to the emergency room due to a CBS, treated previously by a larynx tumour. In this way, it is important to emphasize that all of them underwent a total laryngectomy with bilateral cervical neck dissection (6/6 = 100%). This is why, according to the type of larynx procedure, we found a statistical correlation between open surgical procedures and the development of a CBS (*p* = 0.004). However, we did not find statistical correlation between neck dissection and CBS (*p* = 0.151) when we include all of our patients (open and endoscopic laryngeal procedures). Furthermore, it is important to underline that any patient in our series, underwent a radical neck dissection developing a CBS, in this way, we can suggest that selective neck dissection can be related with the appearance of CBS too, maybe not as a main factor, but it could be associated with other treatment strategies or complications (Table 4).

Another factor related to CBS in previous studies is the presence of mobile foreign bodies in the head and neck like tracheostomy tube, nasogastric tubes, or the presence of wet gauzes. In this case wound healing can be interrupted because of chronic irritation and inflammatory response. According to Chen et al. this could explain why those patients with open wounds in the neck require wound care with wet dressing having a 4-fold increased risk of developing carotid blowout.^19^ Nutritional factors have also been related to the risk of CBS, and this can be explained by the less soft tissue coverage, causing the carotid arterial walls to weaken in the cervical region.^20^ Moreover, in their study Chen et al. found a 2-fold increased risk of developing carotid blowout in patients with a BMI of <22.5 kg/M^2^.^19^

The incidence of cerebral complications in patients affected by CBS, up to 87% when hypotension is present at the time of ligation compared to 28% in normotensive patients.^21^ Furthermore, in those patients who survived an acute episode of carotid blowout, the neurological sequela reported was from 16% to 50%.^9^ Moreover, in a recent study, authors found out that patients with carotid blowout underwent surgical intervention had a higher neurologic complication rate and mortality rate when compared with those of patients received endovascular procedures.^19^ In our series 2 (33.3%) patients showed up neurological sequela after bleeding, one of them died in the first 10 days after the initial episode due to a re-bleeding, and the other patient suffered and hemiparesis as a long term sequela.

About the best option to treat this complication on these days, there exists a trend in favour of endovascular techniques. However, recent studies shows that there is no statistical significant difference in technical and hemostatic outcomes between reconstructive and deconstructive endovascular techniques.^8^, ^22^, ^23^ Moreover, other authors suggest that permanent vessel occlusion results in higher immediately cerebral ischaemia, but stent grafting induces potentially delayed complications, such as infections, rebleeding or stent thrombosis.^8^, ^11^

Finally, our study has a number of limitations. Primarily, its retrospective nature and the small sample size can limit the validity of our results. Moreover, we only included patients treated by larynx tumours, and this kind of complications can affect all those patients treated by head and neck tumours. In this way, a prospective study comparing the results of different types of treatments could be necessary.

## Conclusion

Carotid blowout syndrome (CBS) is an uncommon complication in patients treated by larynx tumours. According to our results, patients underwent radiotherapy and patients treated with open surgical procedures with pharyngeal opening, have a major risk to develop a CBS. In this way is necessary to trying to predict the risk in all of our patients and take the appropriate actions to prevent this kind of complications.

## Conflicts of interest

The authors declare no conflicts of interest.
